# The Book World of Medicine and Science

**Published:** 1895-06-08

**Authors:** 


### THE BOOK WORLD OF MEDICINE AND SCIENCE.

SUGGESTIONS TO HOSPITAL AND ASYLUM VISITORS. By JOHN S. BILLINGS, M.D., Director of the Hospital of the University of Pennsylvania, and HENRY M. HURD, M.D., Superintendent of Johns Hopkins Hospital, with an introduction by S. WEIR MITCHELL, M.D. (Philadelphia: J. B. Lippincott Company. 1895.)

The hospital visitor is often enough a useless, occasionally even an obnoxious person. He does not know exactly what to look for and what to expect, and according to his personal idiosyncrasy indulges in vague and meaningless praise or ridiculous blame. The position is a difficult and often a thankless one. The visitor is more or less an interloper, an unofficial supervisor, a creature in no way necessary to the working of the institution, yet it depends on himself whether he is to become a welcome guest or an intruder whose presence is resented. It is to be remembered that merely to lavish eulogies is not the way to ensure a welcome. Often nurses as well as patients are glad to have defects noticed and pointed out by a visitor, always presupposing that the complaint is reasonable and made courteously. It is wise for a visitor not to attempt to see and remedy too much at one time; to give nurse or superintendent the impression that you think everything wrong that you see is only to give an impression of cantankerous wrong-headedness that defeats its own end. Nor, though the complaints of patients may sometimes be well founded, is it right to accept them without inquiry, still less, as some people do, to publish them to the world instead of laying them before the officials. Hospital authorities do not take in patients in order to torture them to death, as the wild statements of some critics would make us believe, and they are glad when any flaw in the machinery is brought before their notice, but naturally they wax impatient when they are pestered with complaints which show only that the complainer knows nothing about the matter. For the guidance of those who wish to visit hospitals intelligently and helpfully, Dr. Billings and Dr. Hurd have published their little volume of "Suggestions," which we hope will soon be republished in England. It advises what the visitor ought to inquire into in ward, kitchen, bath-room, laundry, and office, not merely points of cleanliness, order, and accuracy, but the relations between the various officials among themselves, and with regard to their patients. It indicates the spirit in which inquiries should be carried out, and advises as to the literature from which a convenient knowledge of hospital requirements may be gained. It should be in the hands of all who interest themselves in our medical charities.

DWELLING HOUSES: THEIR SANITARY CONSTRUCTION AND ARRANGEMENTS. By W. H. CORFIELD, M.A., M.D. Oxon., F.R.C.P.Lond., Professor of Hygiene and Public Health in University College, London, Medical Officer of Health for St. George's, Hanover Square, &c., &c. Third edition, with illustrations. (London: H. K. Lewis, 136, Gower Street, W.C. Price 3s. 6d.)

Dr. Corfield's book well deserves its popularity. It is complete and yet concise. Written by a specialist, it is yet adapted to the intelligence of any householder, and will enable him to decide with fair accuracy the merits and defects of his house, and the appliances best adapted to the remedying of the latter. This is a thing to be grateful for, since in these matters most of us are, in our ignorance and helplessness, completely at the mercy of the plumber, who is often not as wise, occasionally not as honest, as anyone ought to be who is so largely responsible not merely for our comfort but our health.

FOOD AND DRINK RATIONALLY DISCUSSED. By THOMAS DUTTON, M.D.Durham, M.R.C.P.Edin., author of "Indigestion, &c., Clearly Explained, Treated, and Dieted," &c., &c. (London: Henry Kimpton, 82, High Holborn, W.C., and Hirschfield Brothers, 22 and 24, Breams Buildings, Fetter Lane, E.C. Price 2s.)

Dr. Dutton's book deserves its name. While it contains nothing that is very original on the subject of diet, it is thoroughly reasonable and practical. The author has no "issm." He does not deny the importance of gratifying the palate as well as satisfying the stomach, though he protests against over-indulgence in every form. This little volume forms a pleasant guide to gastronomy, which will help the dyspeptic to a wise choice in food, and need not frighten the epicure because it happens to be written by a doctor.

